# Combined biochemical profiling and DNA sequencing in the expanded newborn screening for inherited metabolic diseases: the experience in an Italian reference center

**DOI:** 10.1186/s13023-025-03546-1

**Published:** 2025-01-24

**Authors:** Simona Fecarotta, Lorenzo Vaccaro, Alessandra Verde, Marianna Alagia, Alessandro Rossi, Chiara Colantuono, Maria Teresa Cacciapuoti, Patrizia Annunziata, Sara Riccardo, Antonio Grimaldi, Tonya Fusco, Rosa De Santis, Fernando Barretta, Lucia Albano, Daniela Crisci, Fabiana Vallone, Antonietta Tarallo, Marcella Cesana, Nicola Brunetti-Pierri, Giulia Frisso, Margherita Ruoppolo, Davide Cacchiarelli, Giancarlo Parenti

**Affiliations:** 1 Department of Translational Medicine, University of Naples “Federico II”, Naples, Italy; 2 Azienda Ospedaliera Universitaria “Federico II”, Naples, Italy; 3 European Reference Network for Hereditary Metabolic Diseases, Naples, Italy; 4 Telethon Institute of Genetics and Medicine (TIGEM), Armenise/Harvard Laboratory of Integrative Genomics, Pozzuoli, Italy; 5 Telethon Institute of Genetics and Medicine (TIGEM), Pozzuoli, Italy; 6 NEGEDIA Srl, Pozzuoli, Naples, Italy; 7 Department of Molecular Medicine and Medical Biotechnology, University of Naples “Federico II”, Naples, Italy; 8 CEINGE Advanced Biotechnologies Franco Salvatore, Naples, Italy; 9 Department of Advanced Biomedical Sciences, University of Naples “Federico II”, Naples, Italy; 10 Genomics and Experimental Medicine Program, Scuola Superiore Meridionale (SSM, School of Advanced Studies), Naples, Italy

**Keywords:** Newborn screening (NBS), Inherited metabolic disorders (IMDs), Whole exome sequencing (WES), Next-generation sequencing (NGS)

## Abstract

**Background:**

Newborn screening (NBS) programs have significantly improved the health and outcomes of patients with inherited metabolic disorders (IMDs). Methods based on liquid chromatography/mass spectrometry (LC–MS/MS) analysis are viewed worldwide as the gold standard procedure for the expanded NBS programs for these disorders. Advanced molecular technologies point to genomic sequencing as an alternative and feasible strategy for the screening of genetic diseases, including IMDs. However, each of the two approaches has potential limitations when used as a first-tier analysis. In this study, we tested a workflow-based parallel biochemical and sequencing analyses to determine whether this approach could improve the diagnostic outcome.

**Results:**

For each patient identified by LC–MS/MS as positive, we performed both the biochemical confirmatory tests and next-generation sequencing (NGS) procedures from the same Dried Blood Spot (DBS). NGS analysis was based on applying Exome Sequencing libraries, limiting the analysis to 105 actionable genes involved in IMDs. This allows overtaking the actual limitations of NBS on DBS, enhancing our capacity to identify variants that can drive a genetic disease. Through this approach, we could reach 100% of cases solved, with 37.9% of cases (41/108) for which the combination of the biochemical and NGS analysis was indispensable for a correct diagnosis. In total, we could identify 17 affected, 34 false positives, 12 individuals referred to us for maternal conditions. In 45 newborns the molecular analysis showed heterozygosity for mutations in one or more of the genes analyzed, with results compatible with the biochemical profile indicative of NBS positivity.

**Conclusions:**

In this study, we validated the performance of the proposed workflow. The advantage of this approach is limiting molecular analysis only to positive newborns and using a restricted panel of 105 genes relevant for the expanded NBS, with a 100% rate of diagnosis and potential reduction of the costs related to NBS procedures and reduced impact on patients and families.

**Supplementary Information:**

The online version contains supplementary material available at 10.1186/s13023-025-03546-1.

## Background

Newborn screening (NBS) programs for inherited metabolic disorders (IMDs) are excellent examples of how technological advancements have driven public health policies and improved patient care and outcomes.

After the pivotal studies by Robert Guthrie [1] in the early 1960s that paved the way for the first NBS program for a single IMD (i.e., phenylketonuria) the introduction of technologies based on liquid chromatography-tandem mass spectrometry (LC–MS/MS) in the 1990s resulted in substantial progress of NBS programs. LC–MS/MS can detect multiple metabolites with a single analysis on dried blood spot (DBS) samples and identify several IMDs. Thus, over the past 30 years, the LC–MS/MS-based screening for actionable, early onset, and severe disorders was successfully adopted in most developed countries and translated into significant improvements in patient’s health and quality of life.

However, despite technological advancements, NBS programs need further optimization and standardization of procedures. Much attention has been paid to the number of disorders to be included in NBS panels. It has become evident that there are great disparities in the number of disorders that are included in NBS programs across countries and continents [2]. An evaluation algorithm was proposed to evaluate disorders to be recommended for NBS across Europe, assigning an objective score to each disorder [3, 4].

Also, NBS programs remain associated with important challenges such as those related to non-specific analytes, false positives in carriers or from maternal diseases, and inconclusive results even after a thorough biochemical evaluation. In the context of positive results in NBS, there is often a need to interrogate genomic data as a complement to biochemical data.

Next-Generation Sequencing (NGS) is a valuable diagnostic tool to address critical issues in NBS and to screen for genetic diseases, including IMDs [5–7]. However, genomic sequencing also holds important challenges when used as a first-line approach. For example, once a variant in a disease-causing gene has been identified, there is a need to confirm the diagnosis with biochemistry, particularly for variants of uncertain significance (VUS) or apparent heterozygosity.

The scope of our study was to evaluate the most convenient approach to expanded NBS for IMDs, exploiting the concomitant and integrated use of biochemical and sequencing analyses. To this purpose, we analyzed 108 consecutive neonates referred to our center in the Campania region, Italy for IMDs included in the Italian expanded NBS panel. In these cases, biochemical and sequencing analyses of a set of 105 IMD-causing genes were performed in parallel, starting from a single DBS sample.

## Methods

### Study design

In the Campania Region (Italy), the regional decree n. 30/2019 regulates the procedures for the expanded NBS. The decree mandates that NBS and biochemical confirmatory tests are performed at CEINGE Biotecnologie Avanzate, Naples, in collaboration with the Department of Laboratory Medicine, Federico II University Hospital, Naples. According to this decree, the unit of Pediatric Metabolic Diseases, Federico II University Hospital, Naples is the clinical center for positive newborns all IMDs included in the Italian screening panel [8], except for all forms of hyperphenylalaninemias (phenylalanine hydroxylase deficiencies, defects of biopterin regeneration/biosynthesis), that in Campania Region are referred to another hospital in Naples and are thus not included in this study.

The study received ethical approval from the local ethical committee (Campania 3) for GENOMED study (nr. 3/2023).

### Newborn screening

The NBS in Italy is regulated by the national law 167/2016 [9]. The list of conditions that are screened for according to this law is reported in Table S1. The adopted procedures are described in detail in Ruoppolo et al [8]. In brief, the recommended time for blood sampling is between 48 and 72 h after birth. In low-birth weight infants (< 1800) and in preterm infants (< 37 gestational weeks) blood sample collection is repeated in the first month of life at 15 and at 30 days. Sample shipment to the screening laboratory is carried out by a courier service at controlled temperature, which enables delivery of DBS samples 24–48 h after collection. Newborns’ personal information is entered into the laboratory informatic system by the birth center personnel through a web connection interface.

The methodologies for LC–MS/MS analysis have been previously described [10]. Briefly, metabolites extracted from DBS are derivatized to butyl esters with HCl in N-butanol and LC–MS/MS is carried out by employing 4500 LC–MS/MS systems manufactured by AB Sciex (Toronto, Canada).

Second tier-tests are used to minimize false positives and avoid unnecessary recalls.

The methodologies for biotinidase activity assay [11] and total galactose assay [12] have been previously described.

### Confirmation testing

Biochemical confirmation of positive cases is made by testing plasma amino acids and acylcarnitines, urinary organic acids, urinary orotic acid, plasma homocysteine and succinylacetone. Organic acids are extracted from urine and analyzed by GC–MS analysis as previously described [10]. Homocysteine and plasma vitamin B12 levels are measured according to standard routine methodologies [13] in patients with suspected methylmalonic acidurias to rule out a maternal deficiency and in newborns referred for abnormal methionine levels.

Enzymatic assays are mainly performed to confirm disease severity in some fatty acid oxidation disorders (i.e., MCAD and VLCAD deficiency). The measurement of VLCAD residual activity is achieved in lymphocytes by assaying the oxidation rate of palmitoyl-CoA. The measurement of medium-chain acyl-CoA dehydrogenase (MCAD) function is measured by the oxidation rate of octanoyl-CoA. Both methods are described in Hesse et al. [14].

Galactose-1-P-uridyltransferase (GALT) activity was assayed according to Yamaguci et al [15].

### Whole exome sequencing (WES)

DNA extracted for DBS underwent Whole Exome Sequencing (WES) as previously described [16]. The downstream analysis was restricted to a virtual panel of 105 genes (Table S2) comprehensive of genes that are responsible for disorders included in the Italian expanded NBS disease panel (Table S1) and genes associated with disorders that are considered for the differential diagnosis. This pipeline has the advantages of reducing the time from DBS extraction, accelerating the bioinformatic analysis and facilitating variant interpretation. Variants found by NGS were confirmed by Sanger sequencing.

### Library prep and sequencing

WES libraries were performed using Agilent SureSelect Human All Exon V8 according to the manufacturer’s instructions. Samples were run on Illumina Novaseq6000 as PE 2 × 150 runs with an average depth of 100 × per sample.

### Bioinformatic workflow

The raw data were analyzed by Next Generation Diagnostics srl NEGEDIA Exome pipeline (v1.0) which involves a cleaning step by UMI removal, quality filtering and trimming, alignment to the reference genome, removal of duplicate reads and variant calling [17–20]. Variants were finally annotated by the Ensembl Variant Effect Predictor (VEP) tool (v. 104). Annotated variants were filtered by exonic (+− 10 nt) variants, population frequencies <  = 1% and coverage of alternative base >  = 10x. Filtered variants classification was performed using Varsome Clinical [21], following American College of Medical Genetics and Genomics (ACMG) rules.

### Sanger confirmation

Confirmation of NGS data by Sanger sequencing according to standard published procedures [22].

## Results

### Study design

**Fig. 1:**
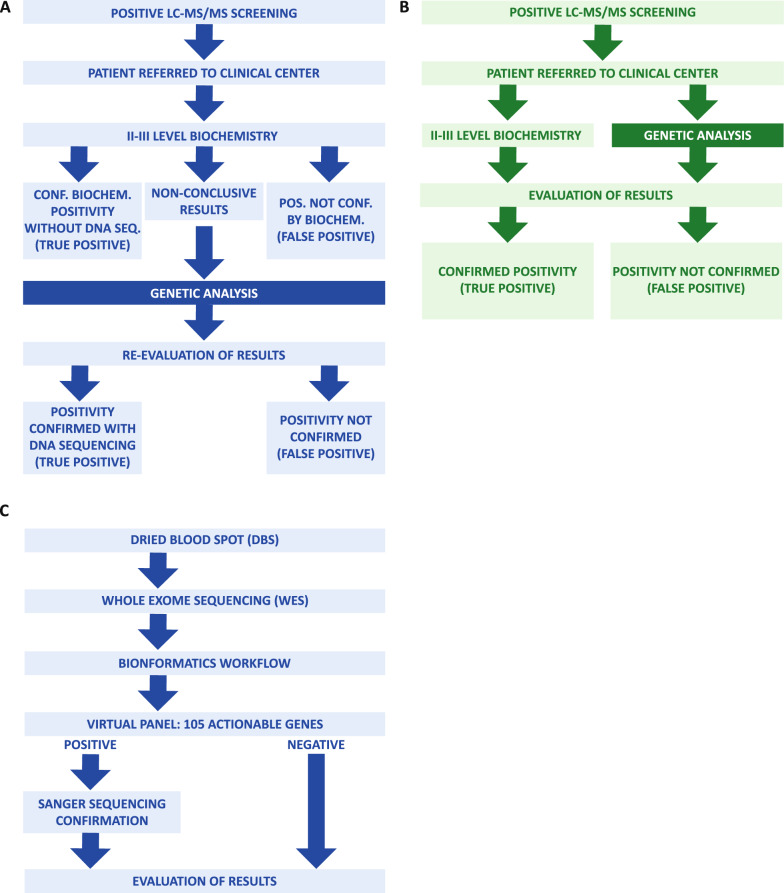
**A** standard procedures defined by Campania regional decree n. 30. Positive newborns are referred to the unit of Pediatric Metabolic Diseases for clinical evaluation and for confirmatory biochemical tests and, after evaluation of the results, molecular characterization. **B** The procedure followed in 108 consecutive newborns referred to us for NBS positivity. In these cases, biochemical and molecular analyses were run in parallel. While in the standard approach the molecular analysis is used as a confirmatory test after the biochemical evaluation, in our study molecular analysis was run in parallel with the biochemical testing. **C** Workflow of NGS and bioinformatic analysis. When both biochemical and molecular data were available, unless a situation of emergency required immediate evaluation and intervention, individual cases were assessed and discussed by a team of metabolic pediatricians and laboratory staff at the end of the procedure

Following standard procedures mandated by the national law 167/2016 and adopted by the Campania Region, NBS is routinely performed on DBS from live births. Newborns that test positive are referred through an informatic platform to the unit of Pediatric Metabolic Diseases, that arranges for a clinical evaluation within 2 working days. At the time of clinical evaluation, confirmatory biochemical tests are performed. After evaluating the results (particularly in cases where biochemical information is insufficient for a precise diagnosis), genetic characterization is performed as complementary or confirmatory analysis (Fig. 1A).

For the present pilot study, from July 2022-November 2022, in 108 consecutive newborns referred to us for NBS-positivity, we adopted a procedure based on combined biochemical and genetic analysis directly from the DBS (Fig. 1B), obtained at the time of the first clinical evaluation at the unit of Pediatric Metabolic Diseases. This change in the diagnostic workflow did not require submission of the study protocol and approval from an Institutional Review Board, as the current regional legislation assigns to the clinical center the decision and the responsibility for all procedures that are deemed necessary for the definition of the diagnosis. The patient’s parents or legal guardians were informed about the procedure to be followed and provided their consent.

### Study results

To speed up the NGS analysis, we developed an optimized workflow in which the analysis of whole exome sequencing was accelerated by the alignment on a virtual gene panel composed by 105 actionable genes causative of IMD (Fig. 1C and Tables S1–S2).

The results of the screening, together with those of the confirmatory biochemical analyses and of molecular analysis are reported in detail for each newborn in Table S3.

Among the 108 patients studied, by using combined and parallel biochemical and sequencing analyses we identified 17 newborns affected by one of the IMDs included in the Italian expanded NBS panel. Specifically, we found 5 patients affected by defects of galactose metabolism (1 affected by galactose epimerase deficiency, ID 137; 4 with *GALT* Duarte variants (compound heterozygote for a Duarte allele in association with pathogenic variants or with variant of uncertain significance—VUS, ID 033, 053, 064, 069); 4 by defects of cobalamin metabolism (ID 048, CblX; ID 068 and 125, Imerlsund-Grasbeck syndrome; ID 086, transient methylmalonic aciduria due to transcobalamin receptor defect); 3 by fatty acid oxidation disorders (ID 118 very-long-chain Acyl-CoA-dehydrogenase deficiency, VLCADD; ID 119 carnitine uptake deficiency, CUD; ID 129 medium-chain Acyl-CoA-dehydrogenase deficiency MCADD); 1 by organic acidemias (ID 102, 3-methylcrotonylglycinuria); 3 by defects of amino acid metabolism (ID 094 hypermethioninemia; ID 120 and 146 Hawkinsinuria) and 1 patient affected by a urea cycle disorder (ID 076, Citrullinemia); (summarized in Table 1).

**Table 1:** Comprehensive table showing the number of individuals prioritized for each class of disorder (rows) and for observational categories (columns)

Group of diseases	Nr. of patients	Nr. of patients diagnosed with biochemistry alone	Nr. of patients diagnosed with the combination of biochemistry and NGS	Homozygous or compound variants	Heterozygous variants	False positives	Maternal conditions
Galactosemia and galactose metabolism-related disorders	26	19	7	5	12	9	0
MMA and related disorders	25	16	9	4	5	4	12
Biotinidase deficiency	9	8	1	0	7	2	0
Increased C5	3	3	0	0	2	1	0
Beta oxidation defects	23	8	15	3	7	13	0
Increased C5OH	8	5	3	1	6	1	0
Abnormal amino acid Profile	14	8	6	4	6	4	0
Total	108	67	41	17	45	34	12

Twelve patients resulted positive because of maternal conditions, all due to maternal vitamin B12 deficiency (ID 037, 038, 039, 050, 115, 065, 066, 071, 092, 108, 143, 126).

In 11 of the patients identified as affected by a specific IMDs (4 patients) or by a maternal condition (7) we also found a condition of heterozygosity for a mutation of an additional gene (for example, ID 137 *GALK1* gene, 068 *CD320*, 125 *SUCLG1*).

Forty-five newborns in whom a diagnosis of IMD was excluded at the end of the study were heterozygotes for mutations in a disease-gene (Table S4) that may have contributed to NBS positivity. In 36 of these cases, we found a condition of heterozygosity for one of the genes included in the panel, in 9 cases we found heterozygosity for two genes (for example both *MCCD1* and *MCCD2* in ID 127; *ETFA* and *ETFB* in ID 100). In all cases the mutations were compatible and related to the abnormal analyte for which the subjects had been referred to the clinical center.

In 11 newborns, one or more variants were incidentally identified in genes unrelated to the metabolites for which the patients had been referred to the clinical center (ID 114, 131, 139, 040, 051, 081, 106, 109, 061, 079, 132). Of course, in these newborns the biochemical profile was not informative (Table S5).

In 34 cases, neither biochemical confirmation nor genetic analysis identified a specific disorder or a condition of heterozygosity.

For each of the cases referred to us, we evaluated whether the traditional approach, exclusively based on biochemical data, would have been sufficient to define a correct diagnosis. This analysis was based on the independent evaluation of three pediatricians with specific expertise in the expanded NBS, followed by a final collegial revision (Table S3), in which all experts reached full agreement on the diagnosis. The experts indicated if the molecular analysis had been critical and indispensable for the diagnosis (indicated in dark grey in Table S3), if it had been a useful support to biochemical data (light gray), or if the biochemistry had been sufficient to define the diagnosis (no color code in Table S3).

**Fig. 2:**
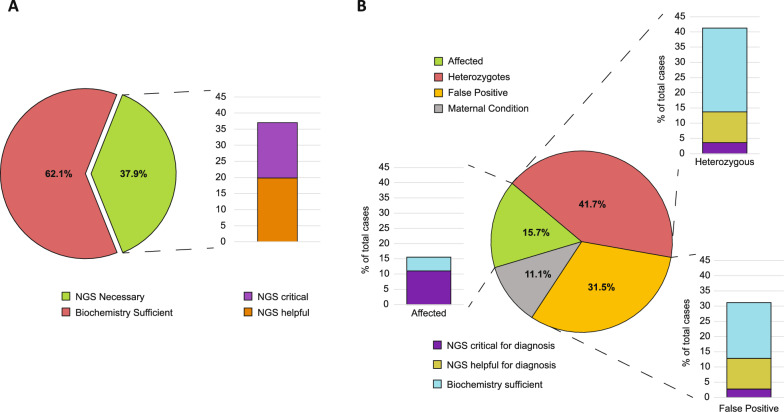
**A** Pie chart representing the amount of case for which biochemical analysis was sufficient to assess the condition and the amount of cases for which NGS was necessary. On the right, barplot showing the percentage of NGS procedures improving or allowing the diagnosis. **B** Pie chart representing the incidence of each type of diagnosis respect to the total of analyzed infants. For each slice, a barplot represents the percentage of cases for which NGS was critical, helpful or biochemistry alone was sufficient

Overall, the combination of traditional biochemical analyses and molecular characterization of patients with the 105-gene panel used in this study was decisive and allowed for a conclusive assessment in 41 cases (37.9%) among 108 studied, resulting critical in 18.5% and helpful in 19.4% of total cases (Fig. 2A). As expected, the contribution of the molecular analysis to a correct assessment of patients and diagnosis varied in the different diseases or group of diseases.

For example, among the 26 newborns referred to the clinical center for increased DBS total galactose considerable overlaps in DBS galactose-1-P-uridyltransferase (GALT) activities did not allow to discriminate between newborns with variable combinations of compound heterozygosity for classic galactosemia due to pathogenic *GALT* gene mutations and Duarte alleles. In addition, in our experience a GALT assay was not promptly available before 4–5 working days. None of these newborns, however, showed clinical signs of galactosemia or required intensive care treatment.

In 1 patient (ID 137) the molecular analysis was essential for a diagnosis of galactose epimerase deficiency, due to *GALE* gene variants. In another patient (ID 049) the molecular findings provided important information to assess a condition of heterozygosity for a *GALE* variant, in the presence of persistent increase of DBS total galactose and low/intermediate GALT activity.

Increased propionyl carnitine (C3) levels in DBS may be due to different genetic conditions, that biochemical analyses alone cannot discriminate. Among the 25 newborns referred to the clinical center for increased C3 with a positive second tier tests, molecular analysis was indispensable in 7 cases (ID 048, 068, 082, 086, 111, 125, 149) to define the diagnosis, while it provided helpful support in clarifying the reason for NBS positivity in other 2 (ID 114, 131). In particular, molecular analysis was critical in patient ID 048 that carried a *HCFC1* gene mutation of uncertain significance and showed persistently increased C3 and modest methylmalonic acid urinary excretion; in this case the combination of biochemical and molecular data suggested a diagnosis of X-linked methylmalonic aciduria and homocystinuria, CblX type, likely with a mild presentation, and indicated further monitoring of the patient. NGS analysis was also critical in patient 086 who carried two *CD320* gene (transcobalamin receptor) mutations and was affected by transient methylmalonic acidemia, TCblR type.

In 3 patients C3 positivity was due to compound heterozygosity for mutations in one or two genes involved in the cobalamin pathway; for example, patient ID 068 (compound heterozygote for *CUBN* and heterozygote for *CD320* gene mutations) and patient ID 125 (compound heterozygote for *CUBN)* that both showed persistent increase in methylmalonic acid urinary excretion.

NGS sequencing supported a correct diagnosis in 16 cases (ID 083, 096, 100, 051, 080, 095,122, 072,141, 130, 107, 081, 119, 099, 104, 106) referred to us for suspected defects of mitochondrial beta oxidation. In this group of patients, the assay of the specific enzyme activities is usually sufficiently informative. However, in the presence of borderline enzyme activities or in conditions in which the defective enzymatic activity is not measurable, a molecular analysis is mandatory to define the situation. In 10 neonates with an acylcarnitine profile at NBS suggestive for defects in which the enzymatic activity is not measurable, such as defective multiple acyl-coA-dehydrogenase deficiency (MADD) or trifunctional protein deficiency/LCHAD, acylcarnitine translocase (CACT)/ carnitine palmitoyl transferase II (CPT-II) or carnitine palmitoyl transferase I (CPT-I), molecular analysis was helpful to define the reason for screening positivity (for example, in patients 083 and 096, both heterozygotes for a mutation of the *ETFB* gene, and in patient 100, heterozygote for a mutation of the *ETFA* and a mutation of *ETFB* gene) or to exclude the diagnosis (ID 051, 080, 095,122, 072,141, 130 all false positives). In 3 cases (ID 099, 104, 106, all false positive) the enzyme assay was not possible for scarce parental compliance. NGS analysis was also fundamental to assess the diagnosis in 3 patients with suspected carnitine uptake defects (CUD), particularly in occasional cases in whom a maternal acylcarnitine profile was not available (ID 107, 081, 119).

In patients that received a diagnosis of a maternal conditions (ID 037, 038, 039, 050, 065, 066, 071, 092, 108, 115, 126, 143, all due to maternal vitamin B12 deficiency) the biochemical analyses were in general considered sufficient for a correct diagnosis. Two newborns (ID 092 and 108) were found to have B12 deficiency, despite their mothers displaying borderline B12 levels (within the low-normal range for a healthy population). In these cases, we hypothesized that maternal B12 levels during pregnancy had been insufficient to prevent increases of C3 and MMA in the newborn period, and to secure an optimal infant cobalamin status during the first 6 months (394 pmol/liter) [23].

However, a negative molecular analysis was of assistance to definitively exclude IMDs. In 7 of these newborns (ID 037, 038, 039, 071, 115, 126, 143) in addition to a maternal vitamin B12 deficiency, the molecular analysis showed one or more heterozygote mutations in disease genes related to the analyte (C3 or C3 ratios) or to the second-tier test (methylmalonic acid, MMA and/or homocysteine, HCY) (Table S5).

The incidence of NGS analysis to each class of diagnosis (affected, heterozygous, false positive or maternal condition) is shown in Fig. 2B

## Discussion

Although LC–MS/MS analysis is worldwide viewed as the gold standard procedure for the expanded NBS programs for IMDs, the growing performance of advanced molecular technologies has pointed to genomic sequencing as an alternative and feasible strategy for the screening of genetic diseases, including IMDs. Pilot projects based on genomic sequencing have already started in some developed countries, for example the BabySeq [24], the Genomic England [25] and the Screen4Care [26] programs. In these pilot studies genomic sequencing has been adopted as a first-tier analysis, in most cases from random newborn cohorts. However, the optimal strategies for the use of this approach and its potential for NBS programs are still debated. Potential drawbacks may be associated with an exclusive genomic approach. For example, as genetic diseases, including IMDs, are mostly rare diseases, it is not infrequent to detect patients that carry VUS which, considered by themselves without the support of biochemical and/or clinical data, have scarce predictive value.

Other studies have proposed a different approach, based on combination of sequencing (as a complementary test) and the traditional LC–MS/MS analysis [27, 28], also in random newborn cohorts. In these studies, the combinatory approach allowed for precise diagnosis definition, with full concordance between biochemical and molecular data.

In the present study we evaluated the effectiveness of a parallel and integrated (both biochemical and molecular) approach to be adopted exclusively in the NBS-positive neonates referred to our center.

This approach was a compromise between the current biochemical methodologies in which DNA sequencing is a second-tier confirmative tests, and NBS based on genomic sequencing in which the analysis is done in all neonates. This allowed us to limit the burden of molecular diagnoses. This is particularly relevant to make the workload related to analyses and interpretation sustainable for clinical centers and in general for the National Health System.

This strategy is even more attractive in view of the expected further expansion of NBS programs for IMDs, such as lysosomal storage diseases that will be soon introduced in Italy. We deemed unnecessary (and probably a waste of resources) to perform molecular screening in patients that had already tested negative at the LC–MS/MS. A restricted panel of actionable genes (rather than whole genome or exome sequencing) was also effective in limiting the risk of incidental findings (and related ethical issues).

This approach proved to be feasible and efficient as we could achieve 100% of diagnoses and identify a total of different variants. Thirty-nine of these variants were VUS (Table S3). Although the interpretation of VUS is often complex and challenging, we found 29 variants (identified in 24 newborns) in genes related to the biomarker or biochemical profile for which newborn screening positivity had been flagged. Thus, also in this respect the combination of biochemical and molecular analysis proved to be helpful in clarifying the reason for the referral, in defining each individual case and in assigning potential clinical significance to the variant.

The approach used in this study was preferable if compared to our previous experience. During the period 2018–2021, 656 neonates of the 178,918 live births in Campania, were referred to our center (1:366 births). Of these, 357 could be diagnosed based exclusively on the basis of biochemical analyses. In a large fraction of cases (299 neonates), however, biochemical data were inconclusive and prompted us to perform a molecular analysis of different sets of genes, each specific for a suspected disease or group of diseases. In 125 of these neonates, we found genotypes compatible with a diagnosis of IMD, in 56 we detected a condition of heterozygosity, 118 were negative to the genetic test. Thus, by this approach in more than 45% of the cases we could achieve a correct definition of the diagnosis only at the end of a sequential, multistep process.

In the present study we immediately proceeded in all patients with the analysis of a NGS panel of genes comprehensive of all genes implicated in the IMDs included in the Italian expanded NBS, thus accelerating the definition of the diagnosis. In all cases, with the combination of biochemical and molecular data, we were able correctly define the diagnosis, including cases in whom the correct assessment of patients was hindered by non-specific analytes, by undefined or overlapping ranges of enzyme activities, by variable combination of pathogenic and mutations or mild variants.

Even though in this initial study we did not reduce substantially the turn-around time for the diagnosis, it is expected that the evolution of sequencing technologies will cut down on time, with obvious consequences on reduction of parental anxiety—a major issue in all NBS programs—and on sequencing costs. It is also possible to speculate that future developments will allow to build combined databases, based on biochemical, molecular, phenotypic data that may have robust predictive value and result into personalized management of patients.

## Conclusions

We validated the combined and parallel use of biochemical and sequencing analyses from DBS in expanded newborn screening program for a panel of inherited metabolic disorders. Due to the wide application of NGS, with progressive potential reduction of its costs, the workflow proposed in this study could become a gold standard in expanded NBS, leading to improved outcome for the infants and potential reduction of impact for the health system and the families.

## Supplementary Information

Supplementary Material 1: Table S1. List of genes analyzed.

Supplementary Material 2: Table S2. List of diseases subjected to NBS in Italy.

Supplementary Material 3: Table S3. Results of biochemical and molecular analyses in DBS from 108 patients.

Supplementary Material 4: Heterozygotes for mutations in one or more of the analyzed genes that were consistent with the biochemical profile indicative of NBS positivity (with no evidence of maternal vitamin B12 deficiency for the MMA/C3 group) (HET, N = 45).

Supplementary Material 5: Additional heterozygosity for mutations in one or more genes associated with the analyte or biochemical profile indicative of NBS positivity in patients with another condition more clearly linked to the screening positivity, which was prioritized for classification (ADDITIONAL HET, N = 11) and Additional heterozygosity for mutations in one or more genes unrelated to the analyte/biochemical profile (INCIDENTAL FINDINGS, N = 11).

Supplementary Material 6: Figure S1. Barplot representing the amount of homozygous or compound (red) and heterozygous (light blue) variants for each class of disorders.

## Data Availability

Sequencing data are not publicly available due to patient privacy. Prioritized variants are listed in Supplementary materials.

## Abbreviations

NBS: Newborn screening
IMDs: Inherited metabolic disorders
LC–MS/MS: Liquid chromatography/mass spectrometry
DBS: Dried blood spot
NGS: Next generation sequencing
VUS: Variants of unknown significance
WES: Whole Exome Sequencing
ACMG: American College of Medical Genetics and Genomics
MADD: Multiple acyl-coA-dehydrogenase deficiency
CACT: Acylcarnitine translocase
CPT-II: Carnitine palmitoyl transferase II
CPT-I: Carnitine palmitoyl transferase I
AC: Serum acylcarnitine profile
AA: Serum amino acid profile
OA: Orotic acid
UOA: Urinary organic acid profile
BTD: Biotinidase
GALT: Galactose-1-P-uridyltransferase
GALE: Galactose epimerase
GALK1: Galactokinase
FP: False positive
HCY: Homocysteine
MMA: Methylmalonic acid
n.a.: Not available
NV: Normal value
PA: Propionic acid
TGAL: Total galactose
Vit B12: Vitamin B12
Wt: Wild-type
