# Supplementary material for: Combined biochemical profiling and DNA sequencing in the expanded newborn screening for inherited metabolic diseases: the experience in an Italian reference center
Source: Orphanet J Rare Dis. 2025 Jan 24;20:38. doi: 10.1186/s13023-025-03546-1 (PMC11762513; doi:10.1186/s13023-025-03546-1)
Supplement: Supplementary file 3 — Supplementary Material 3: Table S3. Results of biochemical and molecular analyses in DBS from 108 patients. [file 13023_2025_3546_MOESM3_ESM.pdf]

Abbreviations

AC serum acylcarnitine profile
AA serum amino acid profile
B benign
OA orotic acid
UOA urinary organic acid profile BTD biotinidase
GALT galactose-1-P-uridyltransferase GALE galactose epimerase
GALK1 galactokinase
FP false positive
HCY homocysteine
LB likely benign
LP likely pathogenic
MMA methylmalonic acid
n.a. not available
NV normal value
P pathogenic
PA propionic acid
TGAL total galactose
Vit B12 vitamin B12
VUS variant of uncertain significance
XLE: Combined concentration of leucine, isoleucine alloisoleucine and hydroxyproline
Wt: wild-type

Color code	Description
DARK GREY	Molecular analysis critical for the definition of the diagnosis
LIGHT GREY	molecular analysis helpful to define the situation but not critical
NO COLOR	biochemistry sufficient for the definition of the diagnosis

GALACTOSEMIA AND GALACTOSE METABOLISM-RELATED DISORDERS

All patients were referred to our clinical center based on confirmed TGAL > 7.0 mg/dL, measured on DBS at newborn screening (data not shown).
Table shows the results of biochemical and molecular analysis performed at the first clinical visit at referral center to clarify the diagnosis

ID	Gender	Analyte	Biochemistry		Genetic analysis	Diagnosis and Comment
			TGAL (NV < 7.0 mg/dL)	GALT activity (NV > 6.8 U/dL)		
033	F	TGAL	12.4	0.9	*GALT* gene*:* c.1057C>T (p.Gln353Ter) (P) / Duarte 2 variant	Biochemistry is insufficient to clarify the diagnosis and the possible consequent need for dietetic therapy, because of persistent high TGAL and very low GALT activity (not immediately suggestive for Duarte galactosemia).<br><br>The proband is a compound for a Duarte variant in combination with a *GALT* pathogenetic mutation.<br><br>Molecular analysis critical to define diagnosis.
034	F	TGAL	7.59	12.4	Negative	FP
036	M	TGAL	8.33	9.7	Negative	FP
044	F	TGAL	3.4	17.4	Negative	FP
049	F	TGAL	9.41	2.8	*GALE* gene: c.304C>G (p.Leu102Val) (VUS) / wt<br><br>*GALT* gene: negative	Biochemistry insufficient to establish the diagnosis, because of persistent increased TGAL and low GALT activity.<br><br>Molecular analysis for *GALT* gene is negative.<br><br>The proband is a *GALE* heterozygote.
053	F	TGAL	10.7	2.5	*GALT* gene: c.983G>A (p.Arg328His) (P) / Duarte 2 variant	The proband is a compound for a Duarte variant in combination with a *GALT* pathogenetic mutation. GALT activity is compatible with this condition.
055	F	TGAL	10.54	6.8	*GALT* gene analysis: negative<br><br>*GALE: c.435_453del (p.Asp145GlufsTer17) (LP) / wt*	Proband referred for high TGAL, but with normal GALT activity. TGAL persistently high.<br><br>Molecular analysis shows heterozygosity for a mutation of *GALE* gene<br><br>Preferable to have a molecular analysis to clarify the situation
056	M	TGAL	3.41	3.8	*GALT* gene: Duarte 2/wt	The proband is a Duarte heterozygote. GALT activity is compatible with this condition.
058	M	TGAL	4.7	9.1	Negative	FP

062	M	TGAL	8.7	3.8	Negative	FP<br><br>The proband is a FP. Because of the low GALT activity, the molecular analysis has been of help in excluding *GALT* gene variants
064	F	TGAL	8.9	0.2	*GALT* gene: c.1057C>T (p.Gln353Ter) (P) + Duarte 2	Biochemistry is insufficient to clarify the diagnosis because of persistent high TGAL and very low GALT activity (not immediately suggestive for Duarte galactosemia).<br><br>The proband is a compound for a Duarte variant in combination with a *GALT* pathogenetic mutation.<br><br>Molecular analysis critical to define diagnosis.
069	M	TGAL	1.76	3.5	*GALT* gene: Duarte 2 + c.628A>G (p.Lys210Glu) (VUS)	The proband is a compound for a Duarte variant in combination with a *GALT* pathogenetic mutation. GALT activity is compatible with this condition.
077	M	TGAL	3.99	14.2	*GALT* gene: Los Angeles variant / wt<br><br>*GALE: c.770A>G (p.Lys257Arg) (B) /c.710-16A>G (LB)*	The proband is a heterozygote for a *GALT* gene Los Angeles variant. GALT activity is normal.<br><br>The proband is also a compound for *GALE* gene pathogenic mutation and a benign variant
085	F	TGAL	2.83	12.5	Negative	FP
088	M	TGAL	3.94	15.2	Negative	FP
093	M	TGAL	3.35	19.5	Negative	FP
097	M	TGAL	6.18	12.8	*GALT* gene: Los Angeles variant / wt<br><br>*GALE* gene: c.266C>T (p.Ala89Val) (VUS)/ wt	The proband is a heterozygote for a *GALT* gene Los Angeles variant. GALT activity is normal.<br><br>The proband is also a heterozygote for a *GALE* variant
101	M	TGAL	15.7	4.9	*GALT* gene: negative<br><br>GALE: c.529-3_529-2del (LP) / wt	The proband is a heterozygote for a *GALE* gene variant<br><br>Reduced GALT activity<br>Persistent high TGAL<br><br>Molecular analysis helpful to define the situation and the reason for screening positivity
110	M	TGAL	6.99	8.6	*GALT* gene: Duarte variant / wt<br><br>GALE: c.559 A>G (p.Asn187Asp) (LP) / wt	The proband is a heterozygote for a *GALT* gene Duarte variant.<br>GALT activity is normal.<br><br>The proband is also a heterozygote for a *GALE* gene mutation

112	M	TGAL	6.02	8.6	*GALT* gene: Duarte variant / wt	The proband is a heterozygote for a *GALT* gene Duarte variant. GALT activity is normal
113	M	TGAL	1.3	10.6	*GALT* gene: Duarte variant / wt	The proband is a heterozygote for a *GALT* gene Duarte variant. GALT activity is normal
134	F	TGAL	2.59	8.3	*GALT* gene: Duarte variant / wt	The proband is a heterozygote for a *GALT* gene Duarte variant. GALT activity is normal
135	F	TGAL	5.22	6.7	*GALT* gene: Duarte variant / wt	The proband is a heterozygote for a *GALT* gene Duarte variant. GALT activity is normal
136	M	TGAL	1,55	22	Negative	FP
137	F	TGAL	13.9	4.7	*GALT* gene: negative<br><br>*GALE* gene: c.770A>G (p.Lys257Arg) (B) (Homozygous)<br><br>*GALK1: c.713T>A (p.Val238Glu)* (VUS) */wt*	The proband is a homozygote for a *GALE* gene variant (currently classified as likely benign)<br><br>The proband is also a heterozygote for a *GALK1* gene mutation<br><br>Persistently high TGAL with intermediate GALT activity.<br><br>Molecular analysis necessary to define diagnosis
148	M	TGAL	6,03	11.4	*GALT* gene: Duarte variant / wt	The proband is a heterozygote for a *GALT* gene Duarte variant. Biochemistry sufficient to exclude classical galactosemia.

MMA AND RELATED DISORDERS

All patients were referred to our clinical center based on increased C3 or C3/C16 or C3/C2 ratio in combination with detected MMA or PA or HCY as second-tier tests, measured on DBS at newborn screening (analyte in table; data non shown).
Table shows the results of biochemical and molecular analysis performed at the first clinical visit at referral center to clarify the diagnosis

ID	Gender	Analyte	Biochemistry	Genetic analysis	Diagnosis and Comment
			AC: C3 (NV <0.65 umol/L)<br>UOA: MMA (NV < 1 mmol/mol Cr)*<br>HCY: NV < 15 umol/L**<br>Vit B12: NV > 197 pg/mL***<br>OA: NV < 1 mmol/mol Cr<br><br>*Urinary MMA < 30 mmol/mol Cr are considered not clinically significant<br>**HCY values > 8 umol/L are suspected for cobalamin-related remethylation disorders (inherited or due to vitamin B12 deficiency)<br><br>*** maternal vit B12 levels < 300 pg/mL could be suggestive for maternal deficiency		
037	M	C3<br>MMA	AC: normal<br>UOA: MMA 65<br>HCY: 14<br>Vit B12: 430<br>Maternal Vit B12: 180	*ACSF3* gene: c.1075G>A (p.Glu359Lys) (P) / wt	Maternal B12 deficiency.<br><br>The patient is also heterozygote for a mutation of the *ACSF3* gene.<br><br>Modest increase in urinary MMA.
038	F	C3<br>MMA	AC: normal<br>UOA: MMA 89<br>HCY: 15.5<br>Vit B12: 128<br>Maternal vit B12: n.a.	*SUCLG1* gene: c.236G>A (p.Gly79Asp) (LB) / wt	Maternal B12 deficiency.<br><br>The patient is also a heterozygote for a mutation of the *SUCLG1* gene.<br><br>Increase in urinary MMA.
039	F	C3/C16<br>MMA<br>HCY	AC: normal<br>UOA: MMA 137<br>AA: normal<br>HCY: 15.4<br>Vit B12: 83<br>Maternal Vit B12: 66.3	*MTHFR: c.3G>C (p.Met1Ile)* (LP) / *c.665C>T (p.Ala222Val)* (B) + *CUBN: c.2452G>A (p.Gly818Arg) (VUS)*	Maternal Vit B12 deficiency.<br><br>The patient is heterozygote for a mutation of the *CUBN* gene. He is also a compound heterozygote for *MTHFR* gene mutation and the thermolabile polymorphism contributing to elevated homocysteine levels
048	M	C3<br>MMA<br>C16<br>C16:1	AC: C3 0.82<br>UOA: MMA 27<br>HCY: 5.4<br>Vit B12: n.a.	*HCFC1: c.4020_4022del (p.Gly1341del) (VUS) / wt*	Modest/borderline increase in C3 and urinary MMA<br><br>Diagnosis: CBLX.

		C18:1	Maternal Vit B12: n.a.		The patient has a mutation in the *HCFC1* gene (likely mild) requiring monitoring.<br><br>Molecular analysis for genes involved in beta oxidation defects is negative.<br><br>Molecular analysis critical to define diagnosis.
050	F	C3<br>C3/C16<br>MMA<br>HCY	AC: normal<br>UOA: MMA 69<br>HCY: 9.8 uM<br>Vit B12: 233<br>Maternal Vit B12: 118 pg/ml	negative	Maternal B12 deficiency.
115	F	C3/C16<br>MMA<br>HCY	AC: normal<br>UOA: MMA 76<br>HCY: 19.5<br>Vit B12: 288<br>Maternal Vit B12: 100	*CBS:* c.833T>C, (p.Ile278Thr) (P) / wt	Maternal B12 deficiency.<br><br>The patient is also a heterozygote for a mutation in *CBS* gene (not critical for diagnosis and follow-up).
065	F	C3/C2<br>MMA	AC: normal<br>UOA: MMA 27<br>HCY: 13.3<br>Vit B12: 107<br>Maternal Vit B12: 128	Negative	Maternal B12 deficiency.
066	F	C3/C16<br>MMA	AC: normal<br>UOA: MMA 46<br>HCY: 6.8<br>Vit B12: 279<br>Maternal Vit B12: 96	Negative	Maternal B12 deficiency.
068	M	C3/C16<br>MMA	AC: normal<br>UOA: MMA 78<br>HCY: n.a.<br>Vit B12: 317<br>Maternal Vit B12: 660	*CUBN* gene: c.10612G>A (p.Glu3538Lys) (VUS) / c.10834C>T (p.Arg3612Trp) (LB)<br><br>*CD320* gene: c.364A>C (p.Lys122Gln) (VUS) / wt	Persistent increase in urinary MMA<br><br>Compound heterozygote for *CUBN* gene mutations (Imerslund-Grasbeck syndrome).<br><br>The proband is also heterozygote for a *CD320* gene mutation (Methylmalonic aciduria, transient, due to transcobalamin receptor defect).<br><br>Molecular analysis necessary to define the situation.
070	F	C3/C16<br>MMA	AC: normal<br>UOA: MMA 24<br>HCY: 6.2<br>Vit B12: 552<br>Maternal Vit B12: n.a.	*CD320* gene: c.262_264del (p.Glu88del) (LB) / wt<br><br>*SUCLG1* gene: c.236G>A	The proband is heterozygote for variants in the *CD320* (Methylmalonic aciduria, transient, due to transcobalamin receptor defect) gene.<br><br>The proband is also heterozygote for a mutation in the *SUCLG1* gene.

				(p.Gly79Asp) (LB) / wt	
071	F	C3<br>C3/C16<br>MMA<br>HCY	AC: normal<br>UOA: MMA 108<br>HCY: 15.3<br>Vit B12: 182<br>Maternal Vit B12: 279	MMACHC: c.848G>C (p.Ter283Serext*1) (VUS) / wt	Maternal B12 deficiency.<br><br>The molecular analysis has also detected a heterozygosity for CblC (not critical for diagnosis and follow-up).
082	F	C3/C16<br>MMA	AC: normal<br>UOA: MMA 78<br>HCY: 5.8<br>Vit B12: 464<br>Maternal Vit B12: n.a.	*ACSF3: c.348G>A, p.(Trp116Ter) (P)* / wt	The patient is a heterozygote for a mutation of the *ACSF3* gene.<br><br>Persistent increase in urinary MMA, with normal Vit B12.<br><br>Molecular analysis critical to define the situation and the reason for screening positivity.
086	M	C3/C2<br>MMA	AC: normal<br>UOA: MMA 55<br>HCY: n.a.<br>Vit B12: 875<br>Maternal B12: 746	*CD320* gene: c.262_264del (p.Glu88del) / c.262_264del (p.Glu88del) (LB)	The patient is homozygous for a variant of the *CD320* gene (Methylmalonic aciduria, transient, due to transcobalamin receptor defect).<br><br>Diagnosis of transient methylmalonic aciduria due to transcobalamin receptor defect, impossible without molecular analysis.<br><br>Persistent increase in urinary MMA, with normal Vit B12.<br><br>Molecular analysis critical for diagnosis
092	M	C3/C16<br>MMA	AC normal<br>UOA: MMA 17<br>HCY: 10.2<br>Vit B12: 122<br>Maternal Vit B12: 343	negative	Maternal B12 deficiency
105	M	C3/C16<br>MMA<br>CPTI	AC: C3 0.75<br>UOA: normal<br>HCY: 4.5<br>Vit B12: 547<br>Maternal Vit B12: n.a.	negative	FP<br><br>Beta oxidation defects were also excluded.
108	F	C3/C16<br>MMA<br>C5/C2<br>C14.1/C16<br>Leu/Phe	AC: normal<br>UOA: n.a<br>HCY: 13.36<br>Vit B12: 149<br>Maternal Vit B12: 312<br>AA: normal	negative	Maternal B12 deficiency.
111	F	C3/C16<br>MMA	AC: C3 1,52<br>UOA: MMA 130<br>HCY: 8.2<br>Vit B12: 349<br>Maternal Vit B12: n.a.	*ABCD4:* c.619C>A, (p.Pro207Thr) (LP)/ wt	Heterozygote for a mutation of the *ABCD4* gene (CblJ).<br><br>Persistent increase in C3 and urinary MMA, with normal Vit B12.<br>HCY levels suspected for cobalamin-related remethylation disorders<br><br>Molecular analysis critical to define the situation and the reason for screening positivity.

114	M	C3/C16<br>MMA	AC: normal<br>UOA: MMA 29<br>HCY: 5<br>Vit B12: 778<br>Maternal Vit B12: n.a.	*MMACHC* gene:<br>c.766_771del<br>(p.Ala256_Pro257del) (VUS) / wt<br><br>*CD320* gene:<br>c.434G>T<br>(p.Cys145Phe)<br>(VUS) / wt<br><br>*CBS* gene:<br>c.833T>C<br>(p.Ile278Thr) (LP)/<br>wt	Heterozygote for a mutation of the *CD320* gene and a mutation of the *MMACHC* gene (CblC)<br><br>Borderline increase of urinary MMA, with normal vit B12 levels<br><br>Molecular analysis helpful to define the reason for screening positivity.<br><br>As an incidental finding the proband is heterozygote for a mutation of the *CBS* gene.
125	F	C3<br>C3/C16<br>MMA	AC: normal<br>UOA: MMA 130<br>HCY: 12.4<br>Vit B12: 275<br>Maternal Vit B12: 124	*CUBN* gene:<br>c.7870A>G<br>(p.Ser2624Gly)<br>(VUS) / c.8134C>G<br>(p.Leu2712Val)<br>(VUS)<br><br>*SUCLG1* gene:<br>c.236G>A<br>(p.Gly79Asp) (LB)<br>/wt	Low/borderline plasma vitamin B12 levels, with increased urinary MMA and suspected levels of HCY<br><br>The patient has not been classified as maternal B12 deficiency because he is a compound heterozygote for two mutations of the *CUBN* gene (Imerslund-Grasbeck syndrome)<br><br>The molecular analysis has been important to clarify the diagnosis.<br><br>The patient is also a heterozygote for a variant of the *SUCLG1* gene.
90	F	C3/C2<br>C3/C16<br>C5/C2<br>Met<br>Arg	AC: normal<br>AA: normal<br>UOA: normal<br>OA: normal	negative	FP
131	F	C3/C16<br>MMA<br>C14:1<br>C16	AC: normal<br>UOA: MMA 30<br>HCY: n.a<br>Vit B12: 885<br>Maternal B12 vitamin: n.a.	*HADHB* c.635C>T<br>(p.Pro212Leu)<br>(VUS) / wt	FP<br><br>Borderline increase of urinary MMA with normal Vit B12 levels. HCY not available.<br><br>Molecular analysis helpful to exclude diagnosis.<br><br>As an incidental finding the proband is a heterozygote for a mutation of the *HADHB* gene.
142	F	C3/C16<br>MMA<br>Arg	AC: normal<br>AA: normal<br>OA: 4<br>UOA: MMA 14<br>HCY: 5.2<br>Vit B12: 298<br>Maternal B12 vitamin: n.a	*LMBRD1*:<br>c.581C>A<br>(p.Ser194Ter) (LP) /<br>wt	The proband is a heterozygote for a mutation of the *LMBRD1* gene (CblF).

143	F	C3/C2<br>C3/C16<br>C3/C4<br>MMA<br>HCY	AC: normal<br>UOA: MMA 50<br>HCY 27<br>Vit B12: 83<br>Maternal Vit B12: 136	*SUCLA2*: c.110T>G (p.Leu37Trp) (B) / wt<br><br>*CBS* gene: c.833T>C (p.Ile278Thr) (LP) / wt	Maternal B12 deficiency.<br><br>The proband is also a heterozygote for a variant of the *SUCLA2* gene and for the common *CBS* gene variant c.833T>C
149	M	C3/C16<br>MMA<br>CPT1<br>Arg	Biochemistry not available because of severe anemia. Only molecular analysis has been performed.	negative	FP<br><br>In this case molecular analysis has been critical to exclude an inborn metabolic disease in the absence of biochemical data
126	M	C3<br>MMA<br>HCY	AC: C3 0.7<br>UOA: MMA 114<br>HCY: 12.5<br>Vit B12: 297<br>Maternal Vit B12: 83	*MMADHC* gene: c.87A>C (p.Lys29Asn) (B) / wt	Maternal B12 deficiency.<br><br>The molecular analysis has also detected a heterozygosity for CblD (not critical for diagnosis and follow-up)

BIOTINIDASE DEFICIENCY

All patients were referred to our clinical center based on confirmed BTD activity < 86 U/dL, measured in DBS at newborn screening (data not shown).
Table shows the results of biochemical and molecular analysis performed at the first clinical visit at referral center to clarify the diagnosis.

**ID**	**gender**	**Analyte**	**Biochemistry** BTD activity in DBS (NV > 86 U/dL)	**Genetic analysis**	**Diagnosis and comment**
035	F	BTD	84.76	*BTD* gene: c.908A>G (p.His303Arg) (VUS) / c.1270G>C (p.Asp424His) (B)	Partial biotinidase deficiency. Compound heterozygote for a VUS and a known benign variant (p.Asp424His)<br><br>Biochemical results with BTD activity below the cut-off are sufficient to establish the diagnosis.
040	F	BTD	118.42	*BTD* gene: c.394A>C (p.Thr132Pro) (LP) / wt<br>+<br>SLC25A13: c.1480A>G (p.Ile494Val) (VUS)/ wt	Heterozygote for a *BTD* gene mutation<br><br>The proband is also a heterozygote for a *SLC25A13* gene (citrin deficiency)
047	M	BTD	88.23	*BTD* gene: c.1308A>C (p.Gln436His) (P) / c.1270G>C (p.Asp424His) (B)	Compound heterozygote for a VUS and a known benign variant (p.Asp424His). Borderline BTD activity is suggestive for a partial deficiency.
052	M	BTD	116.59	*BTD* gene: c.908A>G (p.His303Arg) (VUS) / c.1270G>C (p.Asp424His) (P)	Compound heterozygote for a VUS and a known benign variant (p.Asp424His).
054	M	BTD	109.24	*BTD* gene: c.1308A>C (p.Gln436His) (P) / wt	Heterozygote for a *BTD* gene mutation
060	M	BTD	108	BTD gene: c.1270G>C (p.Asp424His) (B)/ c.1270G>C (p.Asp424His) (B)	FP (Homozygote for a benign variant)
063	M	BTD	97.59	*BTD* gene: c.40G>A (p.Gly14Ser) (P) / wt	Heterozygote for a *BTD* gene mutation
075	M	BTD	142	*BTD* gene: c.-185A>G (B) / c.-185A>G (B)	FP (Homozygote for a benign variant)
145	M	BTD	96,5	*BTD* gene: c.1270G>C (p.Asp424His) (B)/	Compound heterozygote for a pathogenic mutations and a known benign variant (p.Asp424His)

				c.1535 C>T (p.Thr512Met) (P)	

INCREASED C5

All patients were referred to our clinical center based on increased C5 or C5/C2, C5/C4, C5/C0, C5/C3ratio, measured in DBS at newborn screening (analyte in table).
Table shows the results of biochemical and molecular analysis performed at the first clinical visit at referral center to clarify the diagnosis

**ID**	**sex**	**Analyte**	**Biochemistry**<br>AC: C5 (NV <0.24 umol/L)<br><br>UOA:<br>3-OH-isovaleric acid (NV < 18 mmol/mol Cr)<br>N-isovalerylglycine (not detectable)<br>2-ethyl-3OH-propionic (NV < 19.9 mmol/mol Cr)<br>2-methylbutyrylglycine ((not detectable)	**Genetic analysis**	**Diagnosis and comment**
041	F	C5 and C5 ratio	AC: normal<br>UOA: normal	negative	FP
089	F	C5 and C5 ratio	AC: normal<br>UOA: normal	*ACADSB* gene*:* c.443C>T(p.Thr148Ile) (P) / wt	The proband is a heterozygote for a mutation of the *ACADSB* gene.
091	F	C5 and C5 ratio	AC: normal<br>UOA: normal	*ACADSB* gene: c.1186A>G (p.Lys396Glu) (VUS) / wt	The proband is a heterozygote for a mutation of the *ACADSB* gene.

BETA OXIDATION DEFECTS

All patients were referred to our clinical center based on decreased free C0 carnitine or increased C0, C4, C6, C8, C10, C14, C14:1, C16, C16:1, C16:1OH, C18, C18:1, C18:1OH, CPT1 ratio, measured in DBS at newborn screening (analyte in table). Table shows the results of biochemical and molecular analysis performed at the first clinical visit at referral center to clarify the diagnosis.

**ID**	**sex**	Analyte	**Biochemistry**	**Genotype**	**Diagnosis and comment**
			AC:<br>C0 (VN: 10-44.7μmol/L)<br>C4 (VN: 0.12-0.42 μmol/L)<br>C6 (VN: 0.04-0.18 μmol/L)<br>C8 (VN: 0.07-0.25 μmol/L)<br>C10 (VN: 0.09-0.43 μmol/L)<br>C14 (VN: 0.03-0.15 μmol/L)<br>C14:1 (VN: 0.02-0.2 μmol/L)<br>C16 (VN: 0.01-0.23 μmol/L)<br>C16:1 (VN: 0.01-0.07 μmol/L)<br>C16:1OH (VN: 0.01-0.05 μmol/L)<br>C18 (VN: 0.01-0.18 μmol/L)<br>C18:1 (VN: 0.02-0.34 μmol/L)<br>C18:1OH (VN: 0.01-0.07 μmol/L)<br><br>UOA:<br>ethylmalonic acid (NV < 6.5 mmol/mol Cr)<br>2-OH sebacic acid (NV: traces)<br>3-OH sebacic acid (NV < 65.5 mmol/mol Cr)<br>adipic acid (NV < 32 mmol/mol Cr)<br>suberic acid (NV < 20 mmol/mol Cr)<br>glutaric acid (NV < 5.3 mmol/mol Cr)<br>2-OH-glutaric acid (NV 5-69.5 mmol/mol Cr)<br><br>Enzymatic activity:<br>MCAD: 4.1 ± 0.8 mU/mg protein<br>VLCAD: 17.7 ± 2.8 mU/mg protein		
051	M	C16<br>C10DC<br>C18:1<br>C18<br>C16:1OH	AC: normal<br>UOA: normal	*BCKDHA*: c.736G>A (p.Ala246Thr) (VUS) / wt	FP<br><br>AC profile at NBS was suggestive for trifunctional protein deficiency/LCHAD (enzymatic activity not available)<br><br>Molecular analysis helpful to exclude diagnosis of inborn error of metabolism.<br><br>As an incidental finding the proband is heterozygote for a mutation of the *BCKDHA* gene.
124	M	C14:1<br>C14:1/C16	AA normal<br>AC: normal<br>UOA: normal	*ACADVL* gene: c.1844G>A (p.Arg615Gln) (B) / wt	The proband is a heterozygote for a variant of the *ACADVL* gene.<br><br>Intermediate ACADVL activity.

		C5/C2<br>Arg	VLCAD activity: 5.7 (32%)		
080	F	C0<br>C18:1<br>C18:2	AC: normal<br>UOA: normal	negative	FP<br><br>Referred for increased C0, C18:1 and C18:2<br><br>AC profile at NBS was suggestive for carnitine acylcarnitine translocase deficiency (CACT) or carnitine palmitoyltransferase II (CPT II) deficiency (enzymatic activity not available).<br><br>Molecular analysis helpful to exclude diagnosis of inborn error of metabolism.
095	M	C14<br>C16<br>C18<br>C18:1<br>C16:1OH<br>C18:1OH	AC: normal<br>UOA: normal	Negative	FP<br><br>AC profile at NBS was suggestive for trifunctional protein deficiency/LCHAD (enzymatic activity not available).<br><br>Molecular analysis helpful to exclude diagnosis of inborn error of metabolism.
096	M	C10:1<br>C14<br>C16<br>C16:1OH<br>C18<br>C18:1<br>C18:2	AC: normal<br>UOA: normal	*ETFB* gene:<br>c.421T>G, p.(Phe141Val) (VUS) / wt	The proband is a heterozygote for a mutation of the *ETFB* gene.<br><br>AC profile at NBS was suggestive for multiple acyl-coA-dehydrogenase deficiency (MADD) (enzymatic activity not available).<br><br>Molecular analysis helpful to define the reason for screening positivity
099	F	C14:1<br>C5DC	AC: normal<br>UOA: normal<br>VLCAD activity: n.a. (impossible to perform venous blood sampling)	Negative	FP<br><br>The molecular analysis has been helpful to exclude VLCAD diagnosis
100	M	C6<br>C8<br>C10<br>C5DC<br>C12<br>C12:1<br>C14<br>C14:1<br>C14:2<br>C18:1OH	AC: normal<br>UOA: normal	ETFA: c.746G>A, p.(Arg249His) (VUS) / wt<br>ETFB gene: c. 58-13 T>C (VUS) / wt	The proband is a heterozygote for a mutation of the *ETFA* and a mutation of *ETFB* gene [multiple acyl-coA-dehydrogenase deficiency (MADD]<br><br>AC profile at NBS was suggestive for multiple acyl-coA-dehydrogenase deficiency (MADD) (enzymatic activity not available)<br><br>Molecular analysis helpful to define the reason for screening positivity
104	M	C14:1<br>C14:1/C2	AC: normal<br>UOA: normal	Negative	FP

			VLCAD activity: n.a. (impossible to perform venous blood sampling)		The molecular analysis has been helpful to exclude VLCAD diagnosis
107	M	C0	AC: C0 10<br>UOA: normal<br>Maternal AC: n.a.	Negative	FP<br><br>Referred for reduced C0.<br><br>Molecular analysis helpful to exclude carnitine uptake deficiency (CUD) in the presence of persistent low/borderline C0 and not available maternal AC.
081	M	C0	AC: C0 7<br>UOA: negative<br>Maternal AC: C0 11	ACADS: c.511C>T, p.(Arg171Trp) (B)/ c.625G>A, p.(Gly209Ser) (B)	FP<br><br>Referred for reduced C0.<br><br>Molecular analysis helpful to exclude carnitine uptake deficiency (CUD) in the presence of persistent low C0 and normal maternal C0 levels.<br><br>As an incidental finding the patients is compound heterozygote for benign variants of the ACADS gene<br><br>The molecular analysis has been important to clarify the diagnosis
116	M	C8<br>C10	AC: C0 10<br>UOA: normal<br>MCADD enzyme activity: 1.5 (36.6%)	ACADM: c.985A>G (p.Lys329Glu) (P) /wt	The proband is a heterozygote for a mutation of the *ACADM* gene.<br><br>Biochemistry sufficient, molecular results consistent with residual activity
118	M	C14:1<br>C14:1/C2<br>C14	AC: C14:1 0,45<br>UOA: normal<br>VLCAD enzyme activity: 3.4 (19.2%)	*ACADVL*: c.865 G>A (p. Gly289Arg) (LP) / c.1700G>A (p.Arg567Gln) (P) / c.308A>G (p.Lys103Arg)	The proband is a compound heterozygote for three mutations of the *ACADVL* gene.<br><br>Biochemistry sufficient, molecular results consistent with residual activity
119	F	C0	AC: C0 5 and multiple long chains-acylcarnitines reduction<br>UOA: normal<br>Maternal AC: n.a.	*SLC22A5*: c.844del (p.Arg282AspfsTer1) (P) / c.952-11T>A (VUS)	The proband is a compound heterozygote for a mutation of the *SLC22A5* gene.<br><br>Referred for reduced C0, persistent low C0 and maternal AC not available<br><br>Molecular analysis critical for diagnosis of carnitine uptake deficiency (CUD)
129	F	C6<br>C8<br>C8/C6<br>C10	AC: C8 0,29<br>UOA: normal<br>MCAD Enzymatic activity: 0.7 (17.1%)	*ACADM*: c.134A>G (p.Gln45Arg) (P) /c.31-8C>G (VUS)	The proband is a compound heterozygote for a mutation of the *ACADM* gene.

		C10:1			Biochemistry sufficient, molecular results consistent with residual activity
121	F	C0<br>C14:1/C16<br>Orn<br>Arg	AC: normal C0<br>UOA: normal<br>Maternal AC: n.a.	*SLC22A5* gene: c.934A>G (p.Ile312Val) (LB) / wt	Heterozygote for a mutation, likely benign, of the *SLC22A5* gene.<br><br>Molecular analysis helpful to define the reason for screening positivity
138	F	C0	AC: normal C0<br>UOA: normal<br>Maternal AC: n.a.	Negative	FP
144	M	C14<br>C14:2<br>C14:1/C2<br>C14:1/C16<br>C14:1/C4<br>C14:1/C5<br>C14:1/C8	AC: normal<br>UOA: normal<br>ACADVL activity: 6.47 (36%)	*ACADVL*: c.896_898del (p.Lys299del) (P) / wt	The proband is a heterozygote for a mutation of the *ACADVL* gene.<br><br>Biochemistry sufficient, molecular results consistent with residual activity
106	F	C14:1/C16<br>C3/C16	AC: negative<br>UOA: negative<br>VLCAD acitivity: n.a. (impossible to perform venous blood sampling)	*HADHA*: c.158G>A (p.Arg53Gln) (VUS) / wt	FP<br><br>The molecular analysis has been helpful to exclude VLCAD diagnosis<br><br>As an incidental finding, the proband is heterozygote for a mutation of the *HADHA* gene
130	M	C4<br>EMA	AC: negative<br>UOA: ethylmalonic acid 17	negative	FP
083	M	C0<br>C4OH<br>C14<br>C16:1OH<br>C18:1<br>C18:2<br>C3/C4	AC: negative<br>UOA: negative	ETFB: c.-15_-14insA (VUS) /WT	Heterozygote for a mutation of the *ETFB* gene.<br><br>Molecular analysis helpful to define the reason for screening positivity
122	M	C0<br>C2<br>C16<br>C16:1<br>C18:2	AC negative<br>UOA: negative	Negative	FP<br><br>Biochemistry of uncertain interpretation with multiple abnormalities.<br><br>Molecular analysis helpful to exclude the diagnosis.
072		C0<br>C3/C16	AC: negative<br>UOA: negative<br>C3 0.69<br>Maternal Vit B12: 1030	Negative	FP

			HCY: 4.4		AC profile at NBS was suggestive for Carnitine palmitoyltransferase 1 (CPT-1) deficiency (enzymatic activity not available)<br><br>Molecular analysis helpful to exclude the diagnosis<br><br>Moreover, in presence of persistently increased C3 and normal Vit B12 molecular analysis was helpful to exclude MMA-related disorders
141		C0<br>CPT1<br>C5/C2<br>C8/C2	AC: negative<br>AA: negative<br>UOA: n.a.	Negative	FP<br><br>AC profile at NBS was suggestive for carnitine palmitoyltransferase 1 (CPT-1) deficiency (enzymatic activity not available)<br><br>Molecular analysis helpful to exclude the diagnosis

INCREASED C5OH

All patients were referred to our clinical center based on increased C5OH, measured in DBS at newborn screening (analyte in table). Table shows the results of biochemical and molecular analysis performed at the first clinical visit at referral center to clarify the diagnosis

ID	gender	Analyte	Biochemistry	Genetic analysis	Diagnosis and comment
			AC: C5OH (NV < 0.13 umol/L)<br><br>UOA:<br>Lactic acid (NV 1-25 mmol/mol Cr)<br>3-OH-isovaleric acid (NV < 18 mmol/mol Cr)<br>Methylcitric acid (NV 0.5-5.2 mmol/mol Cr)<br>3-methylcrothonylglycine (not detectable)<br>3-methylglutaconic acid (NV < 9 mmol/mol Cr)<br>2-methylacetoacetic acid (NV < mmol/mol Cr)<br>Tiglylglycine (not detactable)<br>Acetoacetic acid (NV < 1.5 mmol/mol Cr)<br>3OH-butyrric acid (NV < 38 mmol/mol Cr)<br>BTD activity in DBS (NV > 86 U/dL)		
046	M	C5OH	AC: C5OH 0,51<br>UOA: negative<br>BTD 205,43	*negative*	FP
067	F	C5OH	AC: negative<br>UOA: negative<br>BTD 335,97	*HLCS* gene: c.1135C>T (p.Gln379Ter) (P) / wt<br>+<br>*MCCC1* gene: c.558del (p.Gln186HisfsTer6) (P) / wt	The proband is a heterozygote for a *MCCC1* mutation and for a *HLCS* mutation
073	M	C5OH	AC: negative<br>UOA: negative<br>BTD 302,05	*MCCC1* gene: c.1132C>T (p.Gln378Ter) (P) / wt	The proband is a heterozygote for a *MCCC1* mutation
084	M	C5OH	AC: C5OH 0,62<br>UOA: negative<br>BTD: 309,46	*MCCC1* gene: c.1790dup (p.Tyr597Ter) (P) / wt	The proband is a heterozygote for a *MCCC1* mutation
098	F	C5OH	AC: negative<br>UOA: negative<br>BTD 343,11	*MCCC1* gene*: c*.320A>G (p.Tyr107Cys) (LP) / c.1772G>A(p.Ser591Asn) (B)<br>+<br>MCCC2: c.1657A>G, p.(Ile553Val) (VUS) / wt +	Biochemistry negative, but the proband is a compound heterozygote for *MCCC1* gene mutations (one pathogenic, one benign)<br><br>The proband is also heterozygote and for a *MCCC2* mutation ad for mutation of the *HMGCL* gene

				HMGCL: c.583A>G (p.Met195Val) (VUS) /wt	Molecular analysis critical for the diagnosis.<br>Due to the uncertain significance of the variants, the patient requires monitoring.
102	F	C5OH	AC: negative<br>UOA: negative<br>BTD 313,14	*MCCC2*: c.1015G>A (p.Val339Met)/ c.1015G>A (p.Val339Met) (P)	Biochemistry negative, but the proband is a homozygote for a *MCCC2* gene mutation<br><br>Molecular analysis is critical for the diagnosis
127	F	C5OH	AC: C5OH 0,45<br>UOA: negative<br>BTD 312	*MCCC1* gene:c.1399A>T(p.Ile467Phe) (VUS) / wt + *MCCC2* gene:c.463C>T(p.Arg155Trp) (P) / wt	Persistent positivity of acylcarnitine profile. Organic acids not informative.<br><br>The proband is a heterozygote for a *MCCC1* gene mutation and for a *MCCC2* gene mutation.<br><br>Molecular analysis helpful to define the reason for screening positivity.
140	F	C5OH	AC: negative<br>UOA: negative<br>BTD 219	BTD: c.1270G>C (p.Asp424His) (P) / wt	The proband is a heterozygote for a *BTD* gene mutation

ABNORMAL AMINO ACID PROFILE

All patients were referred to our clinical center based on increased Met, Tyr, Cit, Arg, Orn, Xle, measured in DBS at newborn screening (analyte in table). Table shows the results of biochemical and molecular analysis performed at the first clinical visit at referral center to clarify the diagnosis

**ID**	**Gender**	Analyte	**Biochemistry**<br><br>AA:<br>Met (NV 10-60 umol/L)<br>Tyr (NV 55-147 umol/L)<br>Citr (NV 10-45 umol/L)<br>Arg (NV 6-140 umol/L)<br>Orn (NV 48-211 umol/L)<br>Val (NV 86-190 umol/L)<br>Leu (NV 48-160 umol/L)<br>Ile (NV 26-91 umol/L)<br>OA (NV < 1 mmol/mol Cr)<br>HCY (NV < 15 umol/L)<br>SUAC (spot) (< 1.85 umol/L)	**Genotype**	**Diagnosis comment**
123	F	Met C14:1/ C16	AA: Met 45<br>AC: normal<br>UOA: normal	*MAT1A* gene: c.596G>A (p.Arg199His) (P) / wt	The proband is heterozygote for a mutation of the *MAT1A* gene associated with the autosomal recessive MAT1/3 deficiency.
109	M	Met C5/C2	AA: Met 62<br>AC negative<br>UOA not performed<br>Vit B12 542<br>HCY 5.2	*MAT1A* gene: c.547 C>G (p. Gln183Glu) (VUS) / wt<br><br>*CUBN*: c.2682C>G (p.Asp894Glu) (VUS) /wt	The proband is heterozygote for a mutation of the *MAT1A* gene associated with the autosomal recessive MAT1/3 deficiency.<br><br>As an incidental finding the proband is a heterozygote for a mutation in the *CUBN* gene.
146	F	Tyr	AA: Tyr 186	HPD: c.97G>A (p.Ala33Thr) / c.97G>A (p.Ala33Thr) (B)	Due to homozygosity for this specific variant of the *HPD* gene, the proband is classified as affected by Hawkinsinuria and requires monitoring<br><br>Molecular analysis important for diagnosis
147	F	ARG	AA: normal<br>OA: 6,4	*ARG1:*c.95G>C (p.Arg32Thr) (VUS) / wt	The proband is a heterozygote for a ARG1 gene mutation
133	M	ARG	AA: normal<br>OA: 1,6	negative	FP
132	F	CITR	AA: Citr 81<br>OA: n.a.	ASS1: c.535T>C(p.Trp179Arg) (P) / wt<br>*CBS:* c.833T>C, p.(Ile278Thr) (LP) / wt<br>ACADL: c.1185del, p.(Glu395AspfsTer23) (VUS) / wt	Persistently modest increased Citr.<br><br>The proband is a heterozygote for a ASS1 gene mutation<br><br>Molecular analysis helpful to define the reason for screening positivity

120	M	Tyr	AA: normal	HPD:c.97G>A (p.Ala33Thr) (B) / wt	HPLC amino acid profile negative.<br><br>The proband is heterozygote for a *HPD* gene mutation, associated with dominant Hawkinsinuria.<br><br>Molecular analysis critical for diagnosis
117	M	XLE<br>Orn<br>Arg	AA: Val 222<br>OA: negative<br>UOA: normal	negative	FP
103	M	Citr<br>Met	AA: Met 129<br>Citr 48<br>HCY 9,5	*AHCY* gene: c.367G>A (p.Gly123Arg) (B) / wt	Persistent hypermethioninemia<br><br>The proband is a heterozygote for a mutation of the *AHCY* (S-adenosyl-homocysteine hydrolase) gene<br><br>Molecular analysis helpful to define the reason for screening positivity
094	F	Met	AA: Met 99<br>HCY 10.3	*MAT1A*: c.412A>G(p.Met138Val) (VUS) / wt +<br>*CBS:* c.833T>C, p.(Ile278Thr) (LP) /wt	The proband is a heterozygote for *a MAT1A* gene mutation (likely dominant)<br><br>Molecular analysis is critical for diagnosis.<br><br>The proband is also a heterozygote for a *CBS* gene mutation.
076	M	Citr	AA: citr 1236<br>OA: 2,2	ASS1: c.535T>C(p.Trp179Arg) (P) / wt	Persistent substantial increase of plasma Citrulline. Biochemical data support a diagnosis of citrullinemia type I and are sufficient to define the diagnosis.<br><br>Only a mutation of the *ASS1* gene has been found. Further analysis is in progress.
061	M	Citr	AA: citr 98<br>OA: 1,3	ASS1:c.839-14G>A (VUS) /wt +<br>SLC7A7: c.1130A>G, p.(Asp377Gly) (VUS) / wt +<br>SUCLA2: c.1289G>T, p.(Cys430Phe) (VUS) / wt	Persistently increased Citr.<br><br>The proband is heterozygote for a mutation of the *ASS1* gene.<br><br>Molecular analysis helpful to define the reason for screening positivity<br><br>As an incidental finding the proband is heterozygote for a mutation of the *SLC7A7* gene and *SUCLA2* gene.
079	F	Citr	AA: normal	CPS1:c.1628C>T(p.Ala543Val) (VUS) /wt	FP
139	M	arg<br>orn<br>C3/C16<br>MMA		*PCCA* gene: c.128 A>G (p.Gln43Arg) (VUS) / wt<br><br>*CBS* gene: c.833T>C (p.Ile278Thr) (LP) /wt	FP<br><br>The proband is heterozygote for a mutation of the *PCCA* gene.<br><br>As an incidental finding the proband is also a heterozygote for the common *CBS* gene variant c.833T>C.
