# Supplementary material for: Combined biochemical profiling and DNA sequencing in the expanded newborn screening for inherited metabolic diseases: the experience in an Italian reference center
Source: Orphanet J Rare Dis. 2025 Jan 24;20:38. doi: 10.1186/s13023-025-03546-1 (PMC11762513; doi:10.1186/s13023-025-03546-1)
Supplement: Supplementary file 4 — Supplementary Material 4: Heterozygotes for mutations in one or more of the analyzed genes that were consistent with the biochemical profile indicative of NBS positivity (with no evidence of maternal vitamin B12 deficiency for the MMA/C3 group) (HET, N = 45). [file 13023_2025_3546_MOESM4_ESM.pdf]

Table S4

Heterozygotes for mutations in one or more of the analyzed genes that were consistent with the biochemical profile indicative of NBS positivity (with no evidence of maternal vitamin B12 deficiency for the MMA/C3 group) (HET, N=45)

	**Galactose**		**C3/MMA**		**BTD**		**beta oxidation defects**		**C5**		**C5OH**		**Abnormal aminoacids profile**	
	**ID**	**Gene**	**ID**	**Gene**	**ID**	**gene**	**ID**	**gene**	**ID**	**gene**	**ID**	**C5OH**	**ID**	**gene**
	049	*GALE*	070	*CD320 + SUCLG1*	035	*BTD*	124	*ACADVL*	*089*	*ACADSB*	067	*HLCS + MCCC1*	123	*MAT1A*
	055	*GALE*	082	*ACSF3*	040	*BTD*	096	*ETFB*	*091*	*ACADSB*	073	*MCCC1*	109	*MAT1A*
	056	*GALT* (DUARTE)	111	*ABCD4*	047	*BTD*	100	*ETFA + ETFB*			084	*MCCC1*	147	*ARG1*
	077	*GALT* (LOS ANGELES)+ *GALE*	114	*MMACHC + CD320*	052	*BTD*	116	*ACADM*			098	*MCCC1 + MCCC2+ HMGCL*	132	*ASS1*
	097	*GALT* (LOS ANGELES) + *GALE*	142	*LMBRD1*	054	*BTD*	121	*SLC22A5*			127	*MCCC1 + MCCC2*	103	*AHCY*
	101	*GALE*			063	*BTD*	144	*ACADVL*			140	*BTD*	061	*ASS1*
	110	*GALT* (DUARTE) + *GALE*			145	*BTD*	083	*ETFB*						
	112	*GALT* (DUARTE)												
	113	*GALT* (DUARTE)												
	134	*GALT* (DUARTE)												
	135	*GALT* (DUARTE)												
	148	*GALT* (DUARTE)												
**Number of cases**	**12**		**5**		**7**		**7**		**2**		**6**		**6**	
