# Supplementary material for: Combined biochemical profiling and DNA sequencing in the expanded newborn screening for inherited metabolic diseases: the experience in an Italian reference center
Source: Orphanet J Rare Dis. 2025 Jan 24;20:38. doi: 10.1186/s13023-025-03546-1 (PMC11762513; doi:10.1186/s13023-025-03546-1)
Supplement: Supplementary file 5 — Supplementary Material 5: Additional heterozygosity for mutations in one or more genes associated with the analyte or biochemical profile indicative of NBS positivity in patients with another condition more clearly linked to the screening positivity, which was prioritized for classification (ADDITIONAL HET, N = 11) and Additional heterozygosity for mutations in one or more genes unrelated to the analyte/biochemical profile (INCIDENTAL FINDINGS, N = 11). [file 13023_2025_3546_MOESM5_ESM.pdf]

Table S5

Additional heterozygosity for mutations in one or more genes associated with the analyte or biochemical profile indicative of NBS positivity in patients with another condition more clearly linked to the screening positivity, which was prioritized for classification (ADDITIONAL HET, N=11) and

Additional heterozygosity for mutations in one or more genes unrelated to the analyte/biochemical profile (INCIDENTAL FINDINGS, N=11)

**Analyte/profile indicative of NGS positivity**	**ID**	**Gene considered for the classification**	**Classification**	**Gene for additional Heterozygosity**	
TGAL	137	*GALE*	diagnosis	*GALK1*	Additional Het
C3/MMA	068	*CUBN*	diagnosis	*CD320*	Additional Het
C3/MMA/HCY	125	*CUBN*	diagnosis	*SUCLG1*	Additional Het
Met	094	*MAT1A*	diagnosis	*CBS*	Additional Het
C3/MMA	037	-	maternal B12 deficiency	*ACSF3*	Additional Het
C3/MMA	038	-	maternal B12 deficiency	*SUCLG1*	Additional Het
C3/MMA/HCY	039	-	maternal B12 deficiency	*CUBN* and *MTHFR*	Additional Het
C3/MMA/HCY	115	-	maternal B12 deficiency	*CBS*	Additional Het
C3/MMA/HCY	071	-	maternal B12 deficiency	*MMACHC*	Additional Het
C3/MMA/HCY	143	-	maternal B12 deficiency	*SUCLA2* and *CBS*	Additional Het
C3/MMA/HCY	126	-	maternal B12 deficiency	*MMADHC*	Additional Het
C3/MMA	114	*CD320 + CblC*	Heterozygote	*CBS*	Incidental findings
BTD	040	*BTD*	Heterozygote	*SLC25A13*	Incidental findings
Abnormal aminoacids profile (Met)	109	*MAT1A*	Heterozygote	*CUBN*	Incidental findings
Abnormal aminoacids profile (Citr)	061	*ASS1*	Heteroygote	*SLC7A7 + SUCLA2*	Incidental findings
C3/MMA	131	-	False positive	*HADHA*	Incidental findings
Beta-Oxidation defect (increased C16, C18:1, C18)	051	-	False positive	*BCKDHA*	Incidental findings
Beta-Oxidation defect (low C0)	081	-	False positive	*ACADS*	Incidental findings
Beta-Oxidation defect (increased C14:1/C16)	106	-	False positive	*HADHA*	Incidental findings
Abnormal aminoacids profile (Arg and Orn)	139	-	False positive	*CBS + PPCA*	Incidental findings
Abnormal aminoacids profile (Citr)	079	-	False positive	*CPS1*	Incidental findings
Abnormal aminoacids profile (Citr)	132	*ASS1*	Het	*CBS + ACADVL*	Incidental findings
