# Supplementary figures and images for: Combined biochemical profiling and DNA sequencing in the expanded newborn screening for inherited metabolic diseases: the experience in an Italian reference center
Source: Orphanet J Rare Dis. 2025 Jan 24;20:38. doi: 10.1186/s13023-025-03546-1 (PMC11762513; doi:10.1186/s13023-025-03546-1)

VARIANTS BY CLASS OF DISORDER

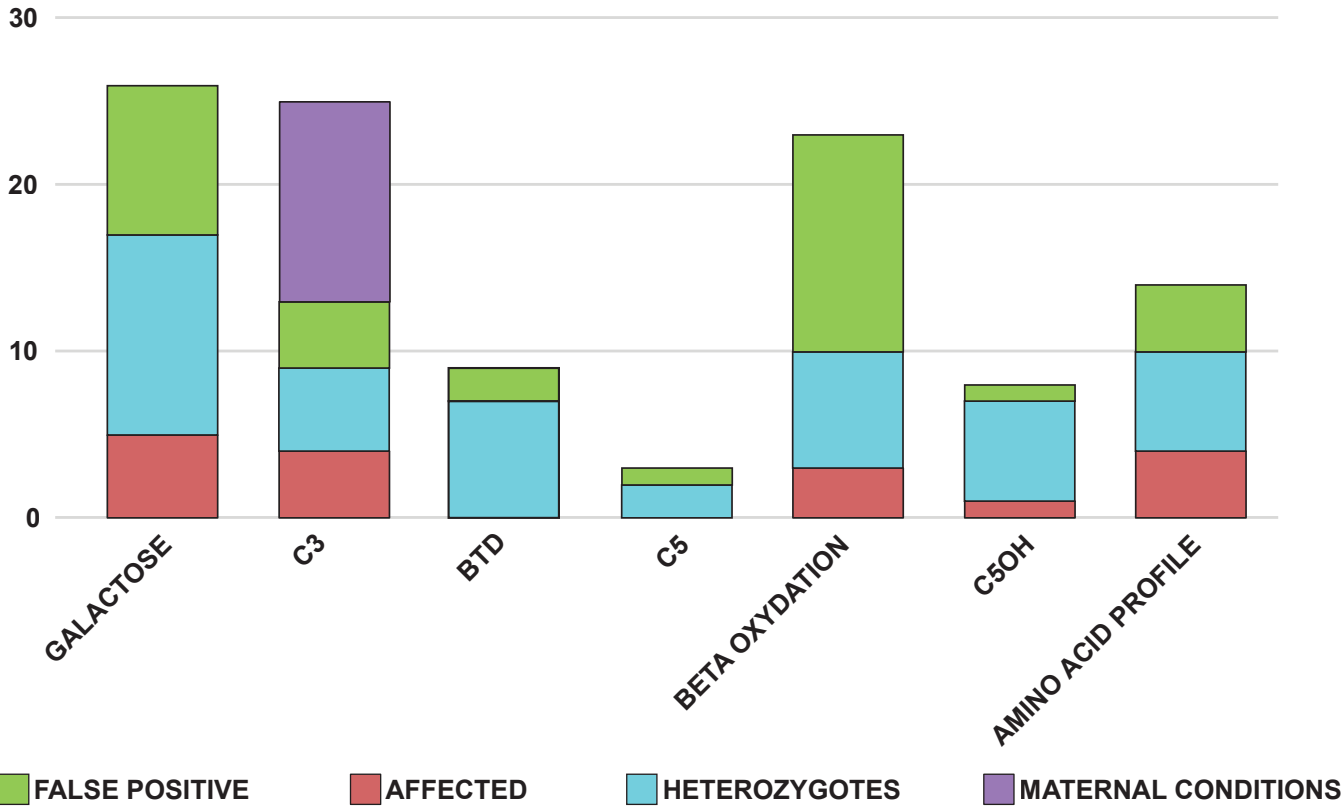

Supplement: Supplementary file 6 — Supplementary Material 6: Figure S1. Barplot representing the amount of homozygous or compound (red) and heterozygous (light blue) variants for each class of disorders. [file 13023_2025_3546_MOESM6_ESM.pdf]
